# Use of compulsory treatment by early career psychiatrists: Findings from an international survey

**DOI:** 10.1192/j.eurpsy.2021.206

**Published:** 2021-08-13

**Authors:** E. Chumakov

**Affiliations:** 1 Day Inpatient Department, St-Petersburg Psychiatric Hospital No 1 named after P.P. Kaschenko, Saint-Petersburg, Russian Federation; 2 Department Of Psychiatry And Addiction, Saint-Petersburg University, Saint-Petersburg, Russian Federation

## Abstract

**Introduction:**

Early Career Psychiatrists (ECPs) are routinely at the front line of clinical practice worldwide, including the use of compulsory measures in psychiatry. However, ECPs practice in this aspect is understudied.

**Objectives:**

The aims of the study were (i) to clarify whether ECPs experience any difficulties in the process of compulsory psychiatric care; and (ii) to find out how ECPs consider compulsory measures in psychiatry.

**Methods:**

An online anonymous survey of ECPs around the world was conducted in July-August 2019. The final sample had 142 psychiatrists (53% female; mean age 32.3±3.1) from 43 countries. Results. 96% of the Early Career Psychiatrists who responded to this survey agree with the continued use of the current legal framework for compulsory psychiatric treatment in their country, either with or without amendments. More than half of the respondents (57%) reported difficulties in providing compulsory psychiatric care due to either challenging interactions with the courts, documentation issues or moral concerns. Over half of the participants (53%) were keen to reform the legal procedures for compulsory psychiatric care in their countries.

**Conclusions:**

The study has shown that there is an agreement among ECPs around the world that legal compulsory psychiatric care procedures are relevant and useful in clinical practice under certain circumstances. As stakeholders, ECPs could be encouraged and involved in adding their own experience and opinions to the debate on the employment of coercion in psychiatry as an ethical and legal issue.

**Disclosure:**

No significant relationships.

